# Retrospective analysis of respiratory isolates post out-of-hospital cardiac arrest to establish choices in empirical antibiotic cover

**DOI:** 10.1186/cc13543

**Published:** 2014-03-17

**Authors:** JL Wong, H Harb, K Bamford, A Ramesh, P Patel

**Affiliations:** 1 Imperial College Healthcare NHS Trust, London, UK

## Introduction

We analysed positive respiratory isolates for antibiotic resistance in our out-of-hospital cardiac arrest (OOHCA) population to establish adequacy of current empirical regimes. Pneumonia commonly complicates OOHCA, with studies suggesting a prevalence up to 48% [1].

## Methods

Patients admitted to the ICU between May 2007 and September 2013 who underwent therapeutic hypothermia post OOHCA were included in this study. Demographics and antibiotic resistance were collected from an electronic database.

## Results

A total of 160 patients were admitted to the ICU post OOHCA. In total, 37.5% (60/160) had no respiratory sample sent within 72 hours of admission and were excluded. Forty per cent (40/100) grew a clinically important isolate. Gram-negative bacteria (GNB) were most frequently isolated (42.5%, 17/40) followed by Gram-positive bacteria (32.5%, 13/40) and mixed bacterial growth (25%, 10/40). *S. aureus (n *= 14) isolates were often resistant to penicillin (92.8%, 13/14 isolates tested) but maintained macrolide (erythromycin 92.8%, 13/14), clindamycin (92.8%, 13/14) and vancomycin (100%, 13/13) sensitivity, if tested. *S. pneumoniae *isolates (*n *= 8) maintained penicillin (87.5%, 7/8), levofloxacin (100%, 6/6), erythromycin (87.5%, 7/8) and vancomycin (100%, 6/6) sensitivity. The most commonly isolated GNB, *H. influenzae,*maintained high-level sensitivity to amoxicillin (81.8%, 9/11) and co- amoxiclav (90.9%, 10/11). Other isolated GNB, however, demonstrated variable resistance to co-amoxiclav (69.2%, 9/13). Isolates were 100% sensitive to pipericillin-tazobactam (17/17), amikacin (12/12) and meropenem (16/16). The British Thoracic Society suggests co-amoxiclav and clarithromycin for first-line treatment of severe community- acquired pneumonia [2]. Based on this, 77.5% (31/40) of our patients would have received adequate cover. If pipericillin-tazobactam replaced co-amoxiclav, 95% (38/40) of our patients would have been treated with appropriate antibiotics.

## Conclusion

Respiratory samples post OOHCA frequently grow potentially pathogenic bacteria but current antibiotic guidelines fail to provide adequate cover in this population.
